# Shift work and metabolic syndrome: A multi-center cross-sectional study on females of reproductive age

**DOI:** 10.3892/br.2019.1211

**Published:** 2019-05-14

**Authors:** Maryam Nikpour, Aram Tirgar, Mahmod Hajiahmadi, Akram Hosseini, Behzad Heidari, Fatemeh Ghaffari, Abbas Ebadi, Fatemeh Nasiri-Amiri, Mojgan Firouzbakht

Biomed Rep 10:311–317, 2019; DOI: 10.3892/br.2019.1205

Subsequently to the publication of this article, the authors have realized that the name and the affliation of the eighth author were presented incorrectly. The name was spelt as ‘Fatemh Nasiri’, whereas the correct spelling should have been ‘Fatemeh Nasiri-**Amiri**’ (as shown above), and the eighth affiliation should have been presented as: Infertility and Health Reproductive Research Center, Health Research Institute, Babol University of Medical Sciences, Babol 47745-47176, Iran. Therefore, the corrected list of authors and affliliations for this paper are shown below.

MARYAM NIKPOUR^1^, ARAM TIRGAR^2^, MAHMOD HAJIAHMADI^3^, AKRAM HOSSEINI^4^, BEHZAD HEIDARI^5^, FATEMEH GHAFFARI^6^, ABBAS EBADI^7^, FATEMEH NASIRI-AMIRI^8^ and MOJGAN FIROUZBAKHT^1^

^1^Student Research Committee; ^2^Social Determinants of Health Research Center; ^3^Department of Biostatistics, Non Communicable Pediatric Disease Research Center; ^4^Clinical Research Development Center, Shahid Beheshti Hospital; ^5^Mobility Impairment Research Center; ^6^Nursing Care Research Center, Health Research Institute, Babol University of Medical Sciences, Babol 47745.47176; ^7^Behavioral Sciences Research Center, Life Style Institute, Faculty of Nursing, Baqiyatallah University of Medical Sciences, Tehran 14359.16471; ^8^Infertility and Health Reproductive Research Center, Health Research Institute, Babol University of Medical Sciences, Babol 47745.47176, Iran.

The authors regret that these errors were not recognized and corrected prior to the publication of the above article, and apologize for any inconvenience caused.

